# TRPV Protein Family—From Mechanosensing to Cancer Invasion

**DOI:** 10.3390/biom11071019

**Published:** 2021-07-13

**Authors:** Tytti Kärki, Sari Tojkander

**Affiliations:** 1 Department of Applied Physics, School of Science, Aalto University, 00076 Espoo, Finland; tytti.karki@aalto.fi; 2 Department of Veterinary Biosciences, Section of Pathology, University of Helsinki, 00014 Helsinki, Finland

**Keywords:** TRPV, mechanosensing, cancer, invasion

## Abstract

Biophysical cues from the cellular microenvironment are detected by mechanosensitive machineries that translate physical signals into biochemical signaling cascades. At the crossroads of extracellular space and cell interior are located several ion channel families, including TRP family proteins, that are triggered by mechanical stimuli and drive intracellular signaling pathways through spatio-temporally controlled Ca^2+^-influx. Mechanosensitive Ca^2+^-channels, therefore, act as critical components in the rapid transmission of physical signals into biologically compatible information to impact crucial processes during development, morphogenesis and regeneration. Given the mechanosensitive nature of many of the TRP family channels, they must also respond to the biophysical changes along the development of several pathophysiological conditions and have also been linked to cancer progression. In this review, we will focus on the TRPV, vanilloid family of TRP proteins, and their connection to cancer progression through their mechanosensitive nature.

## 1. Introduction

All tissues contain specific cellular machineries involved in sensing and converting physical cues into biological responses [1,2]. Mechanosensing hence allows cells to adopt their structure and functions according to the external stimuli, like changes in the composition of the matrix, pressure or shear forces, subsequently regulating all crucial cellular functions and through that the homeostasis of different tissues. Along cancer progression, biophysical properties of the stroma undergo drastic alterations [3,4]. Both stiffness and pressure within the transformed tissues are usually much higher and stromal composition is also commonly altered. Such mechanical cues are recognized by the mechanosensitive machineries and can promote cancer progression f.i. by triggering proliferation and cell migration through several intracellular cascades [5,6]. Additionally, mechanosensing machineries can undergo changes in their function along the neoplastic progression, impairing the ability of the cells to sense and respond normally to the changes in their microenvironment [7,8]. 

Every step along the metastatic progression, including EMT, invasive migration and angiogenesis, is directed by changes in Ca^2+^-homeostasis [9,10]. One of the central players in regulating Ca^2+^-homeostasis and transmitting information about the extracellular biophysical cues is represented by plasma membrane-embedded calcium channels. Therefore, many of the physical features, affecting cancer progression positively, may be mediated by mechanosensitive ion channels that through Ca^2+^ influx favor activation of specific downstream pathways [8,10,11,12,13,14]. Perhaps the most studied Ca^2+^-triggered pathways include calcium/calmodulin-dependent kinase II (CaMKII) and a nuclear factor kappa-light-chain-enhancer of activated B cells (NF-κB) pathways that regulate cell cycle and apoptosis [15,16]. NF-κB protein complex controls the transcription of DNA upon various stresses and is involved in inflammatory and immune responses, regulation of proliferation and cell survival [17,18]. Other important calcium-dependent factors are calcium-dependent cysteine proteases, calpains and the calcium-dependent serine-threonine phosphatase, calcineurin that control f.i. cell cycle, apoptosis and cell migration [19,20,21]. Abnormal activity of mechanosensitive Ca^2+^-channels could therefore play a major role in cancer progression through these pathways or other less studied signaling cascades. Moreover, the expression of such channels is also known to be altered along the neoplastic progression [14,22,23]. The mechanisms behind the abnormal Ca^2+^-channel expression in cancerous tissues are still poorly known but they may be for example linked to the changes in the stromal composition or hormonal status [24,25,26,27]. 

One major group of cell membrane-associated Ca^2+^-channels are formed by the Transient receptor potential (TRP) family proteins that can respond to various extracellular cues, including biochemical compounds, pH, heat, osmolarity and physical stimuli, to trigger activation of specific intracellular cascades through subtle changes in ion influx [28,29]. TRP protein family is composed of over 30 different cationic ion channels that display vital functions in various tissues. This superfamily of proteins has been mostly studied in non-excitable cell types but possesses also important functions in the nervous system [30,31]. Based on the sequence homology, the TRP superfamily is further divided into seven sub-families, more specifically into the TRPV (vanilloid), TRPA (ankyrin), TRPC (canonical), TRPM (melastatin), TRPML (mucolipin), TRPN (NOMPC) and TRPP (polycystin) families [32]. TRPV subfamilies of channel proteins are widely expressed in both non-sensory and sensory cells and display high sequence similarity across different species [33,34,35]. TRPV channels that are capable of sensing physical cues, act in concert with the other cell membrane-linked mechanosensitive structures, including integrin-based cell adhesions, and transmit mechanical signals into a cell readable format [36,37,38,39,40,41,42]. In addition to the extracellular cues, activation of TRPV channels is affected by intracellular signaling, post-translational modifications as well as lipid- and protein-protein interactions, constructing a complex regulatory network [31,43,44]. Upon activation, these channels may then alter membrane potential or concentration of intracellular Ca^2+^ that impacts cellular events. Therefore, deregulation of TRPV and other TRP family channels also plays a major role in the development of several pathophysiological conditions, including various cancers. Mechanosensitive TRPV channels have been linked to cancer progression at least through their ability to both sense and modify mechanically altered microenvironment [45,46,47,48,49], through their association with mechanosensitive Rho GTPases and actin cytoskeleton, leading to altered migratory features [50,51,52,53], as well as due to their role in angiogenesis [54,55,56,57] and cell proliferation [58,59,60]. Many of these channel proteins have also shown potential as therapeutical targets [61,62]. In this short review, we will focus on the unique characteristics of TRPV channels as mechanosensors and the possible connection of their mechanosensitive nature to the progression of various cancers through altered Ca^2+^-signaling.

## 2. TRPV Family—Expression and Biological Functions

TRPs were originally identified in photoreceptors of *Drosophila* [63] and are expressed in a range of species from yeast to humans. TRPs are expressed in a tissue specific manner and they all have a common membrane structure with six transmembrane segments, S1-S6, containing a TRP domain and a loop between S5 and S6, which defines the pore and selectivity of the channel filter [64]. Similar to the voltage-gated potassium channels, TRP tetramers form the functional unit of the cation channels and the ankyrin repeat domain (ARD) is involved in the oligomerizations. TRPV subfamily is constituted by the members TRPV1-6. These are further divided into two subgroups: TRPV1-4 and TRPV5-6. The first group, TRPV1-4, form homo-and heteromeric channels that display mild Ca^2+^-selectivity [20,65,66]. While TRPV5 and TRPV6 can form both homo- and heteromeric channels that are highly selective for Ca^2+^. TRPV channels are structurally similar to the other TRP channels, but they contain additional three to five ankyrin repeat domains in the N-terminus [31]. Despite the high sequence similarity, all of the TRPV family members display specific activation mechanisms and physiological functions.

Many of the TRPV proteins are triggered by multiple stimuli, suggesting that they can participate in the activation of several downstream cascades [33,67,68,69]. TRPV1 is highly expressed in sensory neurons and mainly localized at the plasma membrane [64,70,71]. The channel is activated by heat, pH and compounds, such as capsaicin [31,72] and involved in thermal nociception [41,72,73,74]. Accordingly, TRPV1 KO mice possess impaired heat-evoked pain sensation [41]. While TRPV1 is expressed at the plasma membrane, TRPV2 is mainly localized in the intracellular membranes [75,76]. It is found in the sensory and motor neurons as well as in many non-neuronal cell types [64,77,78,79]. TRPV2 has various physiological roles, from the perception of noxious stimuli to nociception and the importance of its expression has also been demonstrated in the normal function of distinct immune cell types [80,81]. TRPV2 can regulate intracellular calcium homeostasis by acting as a lipid-, thermo- and mechanosensor. Some growth factors, hormones, cytokines and endocannabinoids can also trigger the translocation of TRPV2 from the endosome to the plasma membrane [82]. Additionally, TRPV2 functions can be regulated by Phosphatidylinositol 4,5-bisphosphate (PIP2) and phosphorylation in an extracellular signal-regulated kinase (ERK)-dependent manner [76,83]. Interestingly, TRPV2 is not activated in vivo by vanilloids [70,75,76]. 

TRPV3 is a Ca^2+^-permeable, non-selective cation channel and widely expressed throughout the tissues but especially abundant in various epithelial tissues, including the specialized epithelial cells, keratinocytes of the skin [84,85]. In the skin keratinocytes, TRPV3 can be activated at least by heat and its activation can regulate various downstream functions, including skin barrier formation, wound healing, sensing of temperature, itch and pain [20,71,86,87,88,89,90]. In general, TRPV3 seems to be important for the health of the skin, and loss of TRPV3 function leads to skin inflammations, dermatitis, itchiness and hair loss. Like the expression of TRPV3, the expression of TRPV4 is also very ubiquitous and more prominent in epithelial tissues, where it can induce calcium influx in response to various extracellular cues, such as heat, osmotic changes and mechanical stretching [91,92,93]. TRPV4-mediated Ca^2+^-influx directs various physiological functions in different cell types and has an important role in cell volume regulation [65,94,95,96]. TRPV4−/− mice also display drastic defects in osmoregulation [97,98]. In epithelial tissues, TRPV4 seems to be important for the maintenance of junctional permeability through its impact on adherens junction and tight junction proteins [99,100,101]. In mammary epithelial tissues, TRPV4 regulates the integrity of the cell-cell junctions specifically through the expression of tight junction proteins [102]. TRPV4-mediated calcium influx is also important for the formation of intact cell-cell junctions in skin keratinocytes, where it maintains intercellular barrier function [103]. Besides its important role in tissue integrity, TRPV4 has been connected to several other physiological functions, including its major role in the maintenance of vascular development, tone and permeability [104,105]. TRPV4 has also been linked to the central nervous system and nociceptive response in primary sensory neurons [106,107].

TRPV5 and TRPV6 display a high level of sequence similarity (75% amino acid identity) and are alike in many ways [108]. Both are highly Ca^2+^-selective, 1,25-dihydroxyvitamin D3 responsive and have major roles in the maintenance of Ca^2+^ homeostasis in higher organisms [109,110,111]. TRPV5 is mainly expressed in the kidney, more specifically in the distal convoluted tubules (DCT) and connecting tubules (CNT) [112,113,114], while TRPV6 exhibits a wider expression pattern and is expressed prominently f.i. in the kidney, pancreas, prostate, placenta, and breast [111,115,116]. In Ca^2+^-transporting epithelial tissues, these proteins are localized at the apical membrane and have an important role in intestinal and renal reabsorption [109,113,117]. TRPV6 seems to be constitutively active, and Ca^2+^-influx through this channel depends on the intracellular and extracellular Ca^2+^-balance [118]. The activity of this channel can also be tuned by hormones, that is, estrogen and progesterone, tamoxifen and vitamin D, leading to changes in cell proliferation and survival [119].

TRPV family channels are thus widely expressed in a tissue-specific manner and display numerous functions. The regulation of the activity of these channels is a complex process and impacted by various extracellular and intracellular cues. As demonstrated with TRPV4 [120], at least the N- and C-terminal cytoplasmic domains are important for channel gating. Additionally, processes that affect the oligomerization and trafficking of the TRPVs play a major role in the regulation of these channels and TRPV2, -4 and -5 membrane transportation seems to be dependent on the N-terminal site of the proteins, [121,122,123]. Alternative splice variants of TRPV2 mRNA can also inhibit the translocation of TRPV2, in this way affecting the activity of this channel protein [124]. Regulation of channel trafficking is clearly a complex process and reviewed in more detail f.i. by Doñate-Macián et al., 2019 [125].

## 3. Mechanosensitivity of TRPV Channels

### 3.1. Activation of Mechanosensitive Channels

As already mentioned, TRPV family channels are capable of sensing various extracellular cues to subsequently trigger specific cation-dependent intracellular signaling pathways [126,127]. Initially, the role of these channels in sensing mechanical signals was detected in *Drosophila* and *Caenorhabditis elegans*, displaying TRP gene mutants that had a defective response to the mechanical or osmotic stress [128,129,130,131]. Later, analogous studies were performed in the mammalian and prokaryotic model systems [11,132,133]. Currently, it is known that sensing of physical changes by the channel proteins can take place in two ways: By sensing ”force-from-lipids” or “force-from-filament”, that is, channels can directly respond to membrane stress/stretch/tension or can feel the force through their interaction with cytoskeletal/structural/adhesive components [134,135,136,137,138]. This leads to conformational changes and gating of the channel [139,140,141,142]. The channel activation upon force sensing is very rapid, arising in milliseconds and it insures fast transduction of mechanical stimuli into an ion flux [143]. In case the channels are activated by intracellular mechanosensitive signaling cascades, the activation process is indirect and slower.

### 3.2. Mechanical Stretching and Osmolarity

TRPV channels 1, 2 and 4 seem to be responsive to mechanical stretching and cell size changes due to hypo-osmotic swelling. The activation of TRPV1 by stretch or cell shrinkage has been linked to the mechanical nociception [144,145] and increased osmolarity has been shown to associate with increased Ca^2+^-influx through this channel [146]. The response of TRPV1 to stretch and osmotic stimuli is also dependent on the actin cytoskeleton [147,148]. Both TRPV2 and TRPV4 are also sensitive to membrane stretch [81,125]. In line with that, they are playing an important role in tissues, which are under high mechanical stress, like cardiac and skeletal muscle. In the cardiomyocytes, membrane stretching by hypotonic swelling triggers TRPV2-mediated Ca^2+^-influx [149]. Consequently, TRPV2 has an important role in the maintenance of the structure and synchronized contractility of cardiac muscle [150]. Stretch-dependent activation of TRPV2 has also a function in the regulation of neural circuit formation [77]. In developing neurons, TRPV2 binds actin and has been linked to the actin-dependent axonal outgrowth upon focal mechanical forces [151]. Upon mechanical stimulation, TRPV2 has been shown to rapidly accumulate at the site of the stress [151]. Clustering of TRPV2 at the sites of mechanical forces then leads to reorganization of the actin cytoskeleton, subsequently promoting axonal outgrowth. It seems that strict regulation of axonal out-growth also requires interplay between several other mechanosensitive structures [152]. As the ankyrin repeats of TRPV2 interact with many cytoskeletal proteins and the ankyrin repeats at the N-terminal region of TRPV proteins, are important for their mechanosensitive features, the association of TRPV2 with the cytoskeletal structures may be necessary for its function as a mechanosensor [77,151,153]. The stretch sensitivity of TRPV2 has also been studied f.i. in CHO and HEK293 cell lines. In both of them, altered Ca^2+^-response was detected upon stretching on elastic substrates [77,149,154]. Additionally, studies on rat lung alveolar epithelial type II, ATII, cells showed that the strain-induced, TRPV2-dependent Ca^2+^-influx was dependent on the focal adhesions and actomyosin structures [155], again linking the mechanosensitive activity of TRPV2 to the cytoskeleton. There is also strong evidence that TRPV2 acts as a mechanosensor in the circulatory organs and intestinal tract [78,81]. 

TRPV4 is probably the most studied TRPV family channel in respect of its mechanosensitivity. Its activation has been explored in various tissue types and based on these studies TRPV4 is clearly an important osmo-mechanosensitive channel, responding to variations in membrane stretching upon osmotic changes. Some of the first reports on TRPV4 –/– mice have shown the importance of TRPV4 in pressure sensation and a higher threshold for the response to strong noxious mechanical stimulation [93,97,156]. Early studies on TRPV4 have also shown its prominent expression in the kidney [91,157,158,159,160]. Kidney cells are constantly exposed to extracellular fluids/changes in the osmolarity and TRPV4 in the cortical collecting ductal cells has an important role in the cell volume regulation in an aquaporin-2 and cytoskeleton-dependent manner [161]. Studies in HEK293 or CHO-K1 cells have also shown the activation of TRPV4 by the application of hypotonic solutions [91,157,158] and in primary sensory neurons, hypotonic solutions activate TRPV4 to trigger nociceptors [106]. In line with the study of Galizia et al., 2012 [161], other studies have as well presented evidence for the importance of intact actin cytoskeleton in TRPV4-dependent cell volume regulation [162,163,164,165]. Furthermore, TRPV4 has an important role in the mechanical regulation of calcium homeostasis of the cardiomyocytes, subsequently affecting their contractility but also repair after cardiac infarction [166,167,168]. There are also indications that TRPV4-mediated Ca^2+^-influx regulates the contractility of airway smooth muscle cells and vascular endothelial cells [108,169]. Besides its expression in the artery endothelial cells, TRPV4 can be found in the vascular smooth muscle cells of some arteries [170]. While in these artery smooth muscle cells TRPV4 activation seems to be involved in the regulation of arterial dilation, its upregulation in pulmonary arterial smooth muscle cells has been reported to increase smooth muscle tone and cause pulmonary hypertension in chronically hypoxic rats [171]. Moreover, TRPV4-triggered Ca^2+^-influx has also been studied in urothelial cells, where this channel has been linked to ATP-release upon stretching, hypotonic solutions or intravesicular pressure changes [172,173,174]. Furthermore, recent studies have shown that some TRPV channels can also be triggered by chemical agonists through their ability to sense mechanical perturbations in the plasma membrane [175]. At least with TRPV4, this is linked to the regulation of innate defense mechanisms against bacterial infections [176,177,178].

### 3.3. Activation upon Shear Forces

In addition to membrane-stretching, TRPV channels are also responsive to shear forces. In the cardiovascular system, TRPV4 is prominently expressed in the endothelial cells of larger arteries and arterioles and mediates shear-stress-induced relaxation [179,180,181,182]. This response can be disrupted by specific TRPV4 inhibitors or downregulation of the channel protein. In contrast, TRPV4 activation leads to vasodilation [180]. Moreover, shear stresses have been shown to regulate the clustering and translocation of the TRPV4 channel from the adherens junctions to the basal side of the endothelial cells, in a focal adhesion kinase and integrin α5ß1-dependent manner [183]. Interestingly, both TRPV4 and TRPV6 have been functionally linked to the shear force-sensitive cilia and microvilli. Cilia can sense various mechanical stimuli in distinct organs and TRPV4 seems to be an important component of this mechanosensitive structure [184,185]. TRPV4 has been shown to be associated with the primary cilia, through which it is involved in sensing both hypo-osmotic solutions and mechanical cues, subsequently triggering Ca^2+^-response in ciliated epithelial tissues [186,187]. For instance, in the airway epithelium, TRPV4 is prominently expressed and responsive to the airflow-caused physical shear stresses [188]. Moreover, TRPV4-mediated Ca^2+^-influx can also couple changes in fluid viscosity to the changes in ciliar beat frequency [189], creating a regulatory feedback loop. TRPV6 on the other hand has been linked to microvilli, actin-based membrane protrusions that have an important role in the mechanotransduction within various epithelial tissues [190]. TRPV6 is responding to shear forces in human placental trophoblastic cells and the following Ca^2+^-influx promotes the formation of microvilli in an Akt/Ezrin-dependent manner [190]. TRPV6 could therefore have a major role in microvilli-mediated mechanosensitive functions in various epithelial tissues. 

### 3.4. Stiffness-Sensing

Moreover, there are indications that some TRPV channels can act as stiffness-sensors. In the mouse epidermal keratinocytes, TRPV4 has been shown to play a major role in tissue stiffness-sensing and subsequently regulate YAP/TAZ localization [191,192]. On the other hand, TRPV4 itself mediates collagen matrix assembly, matrix stiffening and promoting fibrosis [193,194], in this way creating a feedback loop in both sensing and creating a mechanically altered cellular environment. Additionally, TRPV6 expression levels seem to be dependent on the stiffness of the underlying matrix, at least in the 2D mammary epithelial cell culture model [45]. 

### 3.5. Sensing Forces through Integrin-Based Adhesions

Integrin-based adhesions act as central sensors of the biophysical changes in the cellular microenvironment [195]. They respond to various physical cues, including stiffness, composition and applied external forces [196]. Integrins and cytoskeletal structures also directly form complexes with TRPV channel proteins and could in this way transmit information on the biophysical changes to subsequently trigger the opening of the ion channels [197]. TRPV4 interacts directly with α2-integrin and the Src tyrosine kinase Lyn [198]. TRPV4 is also known to be triggered through forces applied to β1-integrin [199]. Mechanistically, forces from the cytoplasmic tail of β1-integrin are transmitted through the CD98hc cytoplasmic tail to the TRPV4 ankyrin repeat domains to activate force-induced Ca^2+^-influx [200]. Besides TRPV4, at least TRPV1 is linked to integrins: TRPV1 is expressed together with integrin subunits that can bind fibronectin and TRPV1 translocation to the cell membrane takes place in a fibronectin-dependent manner, playing a role in primary sensory neuron sensitization [201]. Additionally, integrin-TRPV cooperation plays an important role in osmosensation [197]. Most likely, integrins and TRPV channels have many additional interconnections upon various external physical cues and their roles in mechanotransduction should be further investigated. 

### 3.6. Are All the TRPV Channels Mechanosensitive?

While TRPV2 and 4 seem to be clearly mechanoresponsive, the other channels are less studied and there are no clear indications f.i. for the role of TRPV3 or TRPV5 in the mechanotransduction. Additionally, in the study by Higashikawa et al., TRPV3 was not found to be stimulated by mechanical stretching [202]. It has, however, been shown that TRPV3 forms a signaling complex with TGF-α and EGFR and that this complex plays a role in the mechanical skin barrier function [203]. TGF-α and EGFR are also known to regulate mechanotransduction pathways, involved in contractility and cell migration [204]. Furthermore, TRPV1, TRPV3 and TRPV4 ankyrin repeat domains bind ATP and calmodulin (CaM) [205]. CaM is also linked to the regulation of mechanosensitive cascades, affecting cell migration [206]. Besides these, there are other known TRPV channel interactions with unknown consequences and the functional significance of these associations for the cellular mechanotransduction events should be studied in the future. 

## 4. Expression and Activity of TRPV Channels along Cancer Progression

Among other TRP family channels, TRPV channels have been associated with a wide variety of human cancers [58,59,207]. Altered expression of TRP channels has been linked to cancer progression through enhanced cell proliferation, changes in differentiation and impaired cell death, leading to the uncontrolled expansion of the transformed cells [58,59,60,124]. The major Ca^2+^-triggered pathways, affecting the above features, include CaMKII, NF-κB, calpains and calcineurin pathways [15,16,19,20,21]. Besides them, other less studied signaling cascades may affect cancer progression through TRP-triggered Ca^2+^-influx.

Changes in the expression of TRP channel proteins are thought to play a role in the later stages of various cancers—not in the actual carcinogenesis or initial phases in the cancer progression. Mutations in the TRP genes are not common, and mostly either the expression or activity of these channels is deregulated along the progression [12,16,124,208]. TRPV1 is abnormally expressed at least in breast, prostate and urothelial cancers as well as in human papillary thyroid carcinoma and gliomas [209,210,211,212,213,214]. The upregulation of TRPV1 in high-grade prostate and breast cancer samples correlates with the tumor grade [209,210], while downregulation of TRPV1 expression is associated with the progression of urothelial cancers [215]. In androgen-responsive NCaP prostate cancer cells, Capsaicin, a TRPV1 agonist, was found to enhance TRPV1-dependent cell proliferation through Akt and ERK pathways [216]. Cross-talk in between α1D-adrenoceptors and TRPV1 seems to enhance cell proliferation and targeting both TRPV1 and α1D-adrenoceptors could act as a therapeutical choice [217]. In another study, performed with PC3 prostate cancer cells, Capsaicin was, however, found to inhibit proliferation and induce apoptosis [218]. This took place via inhibition of coenzyme Q activity, leading to increased ROS generation and caspase-3 activation. Interestingly, in the MCF-7 breast cancer cell line, both TRPV1 agonists and antagonists significantly reduce cell growth with yet poorly known mechanisms [219]. Therefore, both too high and too low TRPV1 expression, through specific intracellular pathways in a cell type specific-manner, may provide an advantage for the expansion of cancer cells. Other than proliferation, TRPV1 has been associated with cell migration and in human hepatoblastoma HepG2 cells TRPV1-triggered Ca^2+^-influx promotes cell migration [220].

The link between abnormal TRPV2 expression and cancer progression has been widely studied and TRPV2 seems to exhibit oncogenic activity in cancers of the prostate and breast, as well as in esophageal squamous cell carcinoma, leukemia, multiple myeloma and glioblastomas [82,221,222,223]. Overexpression of TRPV2 in urothelial-, prostate-, esophageal squamous cell- and hepatocarcinoma tissues, as well as cell lines of the same cancer types, correlates with advanced disease and metastasis [224,225,226,227,228]. As with other TRP channels, changes in TRPV2-mediated signaling result in uncontrolled proliferation, impaired apoptosis and changes in the migratory features of the cells [82,221,229,230]. In the prostate carcinoma model, trafficking of TRPV2 to the plasma membrane correlated with enhanced cell migration through the phosphoinositide 3-kinase (PI3K) pathway [231]. In bladder cancer cells, TRPV2 also enhanced cell migration and invasion but did not affect cell proliferation in vitro [89,232,233]. In hepatoma and hepatocarcinoma models, TRPV2 may also increase drug resistance [234] and suppression of TRPV2 activity in nude mice xenografts reduced tumor growth and invasion [226]. On the other hand, in glioblastoma cells, stimulation of TRPV2 could sensitize cancer cells to cytotoxic chemotherapeutic agents [235]. Furthermore, Mizuno et al. 2014 reported that lower TRPV2 levels in bladder cancer cells are associated with increased proliferation, again indicating cell-type-specific differences in the role of this TRPV channel during cancer progression [233]. In contrast to TRPV2, TRPV3 has been much less studied but it is a known regulator of growth and survival of the skin cell populations and its overexpression has been associated at least with the proliferation of lung cancer cells [236,237]. As TRPV3 is present in the same signaling complex with EGFR and EGFR activity triggers TRPV3 [203], there is a clear functional interplay in between these factors and it potentially plays a role along cancer progression. 

Abnormal TRPV4 expression is linked to at least gastric, liver, pancreatic, colorectal, lung and breast cancers [59,238,239]. A significant upregulation of TRPV4 has been detected in breast cancer cell lines with the potential to metastasize and its expression seems to increase with tumor grade and size, subsequently correlating with poor survival [240,241,242]. Additionally, TRPV4 has been linked to cell proliferation through the CaMKII pathway and regulation of apoptosis in distinct cancer models [243,244]. The expression of TRPV5 is more restricted, and this TRPV family protein is also less studied in comparison to highly similar TRPV6. The role of TRPV6 has been extensively investigated upon malignant transformation in various tissues and its expression is known to be elevated in many cancers [111,116]. Expression of TRPV6 is upregulated in prostate, breast, colon, esophageal and cervical tumor tissues, as well as in the corresponding tumor cell lines. Increased expression of TRPV6 stimulates the metastasis of cancer cells and confers chemotherapy resistance [116,245,246,247,248]. In at least prostate and breast cancer, high TRPV6 expression is linked to cellular proliferation and invasion through Ca^2+^-dependent pathways and its high levels have been proposed to act as a prognostic factor [119,249,250,251]. Additionally, in prostate cancer, TRPV6 contributes to cell survival and resistance to apoptosis [247]. Distinct TRPV channels thus act as central regulators of the hallmarks of cancer, but they also display clear functional differences in a cancer-type-dependent manner.

## 5. TRPVs and Cancer Progression—Links to Mechanosensitive Pathways

### 5.1. Interplay in between Small Rho GTPases and TRPVs 

Deregulation of TRP channels and altered Ca^2+^ homeostasis have been directly linked to the hallmarks of cancers [60]. Spatio-temporal activation and duration of Ca^2+^-influx determine the activation of specific signaling cascades and transcription factors that guide various cellular processes but also play a role in cancer progression [21,252]. Besides the most studied Ca^2+^-triggered pathways, CaMKII, NF-κB, calpains and calcineurin pathways, also specific mechanosignaling routes may favor cancer progression through TRP-triggered Ca^2+^-influx.

Small GTPases (Rho- and Ras-like) that act as major regulators of the mechanical signaling are linked to cancer progression through their impact on actin dynamics, cell polarity, differentiation and proliferation [204,253,254,255]. Interestingly, there is interplay between TRPs and small GTPases along cancer progression: Many small GTPases interact with calcium-dependent signaling cascades and can trigger Ca^2+^-associated effectors, impact TRP trafficking to the plasma membrane or directly affect the gating and activity of certain TRP channels [50,51,52,53]. Conversely, the activity of small GTPases has a strong association with Ca^2+^-homeostasis [50], suggesting a reciprocal interaction between TRPs and small GTPases. TRPs are associated with Rho-dependent cytoskeletal reorganizations, cellular contractility and cell adhesion turnover, subsequently affecting migratory features of the cancer cells [256,257]. In breast cancer cells, treatment with Rho-kinase inhibitors leads to a reduction in TRPV2 levels [258], indicating a Rho-dependent regulation of TRPV2 activity. Additionally, Rac1 is able to dictate intracellular trafficking of TRPV2 in fibrosarcoma cells [259], implicating a central role of these Rho GTPases in TRPV2 regulation. While in some cancer cell lines TRPV2 seems to have a positive impact on cancer cell migration [223,226,260], the interplay between TRPV2 and RhoA/Rac1 seems to suppress invasion of Fibroblast-like Synoviocytes (FLS cells) [261]. In this model setup, TRPV2 stimulation caused a decrease in RhoA and Rac1 activity, consequently leading to a lower number of contractile actin bundles and cell adhesions as well as inhibition of lamellipodia formation [261]. As Activation of Rac1 through the PI3 pathway leads to TRPV2 translocation to the plasma membrane, subsequently resulting in increased Ca^2+^-triggered invasiveness in some cell types [259] and active TRPV2 seems to inhibit Rac1 [261], there may be a regulatory feedback loop in between these two factors and TRPV2 could display both invasion-promoting or inhibiting features in a cell-type-specific manner.

The role of TRPV4 in cancer metastases has been investigated in several model systems [239,262] and TRPV4 has also been most thoroughly studied with respect to its mechanosensitive character along cancer progression [240,262,263,264]. Like TRPV2, TRPV4 has also been connected to cancers through Rho GTPases that act as major mediators of mechanical signaling. TRPV4 has recently been found to form complexes with RhoA and the TRPV4-mediated Ca^2+^-influx positively affected RhoA activity, subsequently leading to cytoskeletal changes [265]. Additionally, upon growth factor stimulation, TRPV4-mediated Ca^2+^-influx leads to activation of RhoA/ROCK pathway and subsequently causes increased contractility of ECM-modifying fibroblasts [49,166,266,267]. On the other hand, in tumor endothelial cells, overexpression of TRPV4 seems to have an opposite effect on RhoA activity [57]. TRPV4 has also been associated with Rho/ROCK pathway, cytoskeletal changes and invasion in cancer models: In endometrial cancer, TRPV4 promotes metastasis by Rho/ROCK-induced cytoskeletal changes [268]. In line with this, breast cancer models have shown that TRPV4 over-expression impacts cancer cell migration by leading to the higher activity of ROCK-regulated cofilin that promotes actin filament depolymerization [240]. Such cells become mechanically more compliant, enabling transendothelial migration. Additionally, in endometrial cancers, TRPV4 regulates cancer cell invasion through RhoA/ROCK1-dependent cytoskeletal changes [268] and in glioma cancer cells TRPV4 promotes invasion through Akt/Rac1 signaling pathway [269]. Akt pathway is also involved in gastric cancer invasion through, TRPV4-triggered Ca^2+^ influx [264]. Akt itself has been suggested to play a role in the adhesiveness of metastasizing cancer cells in a force-triggered manner [270]. Furthermore, TRPV4 impacts cell-exerted forces in migratory cells and affects dynamics of the trailing adhesions, probably through its interactions with some Rho-responsive focal adhesion proteins and co-operation with other cation channels [271]. Moreover, there are indications that deregulation of TRPV4 could potentiate cancer invasion by affecting scattering of cancer cells: Adherens junctions seem to be regulated through TRPV4-mediated activation of Rho GTPases that induce reorganization of actin-based structures along junction formation [272]. High TRPV4 levels thus favor breast cancer metastasis by regulating cell-cell contacts, actin-dependent cell compliance, cell migration and extravasation through the AKT-E-cadherin signaling [240,241]. 

### 5.2. TRPV-Linked Epithelial Mesenchymal Transition and Stiffness of the Microenvironment

Loss of E-cadherin and intact cell-ell junctions are important features in the process of epithelial mesenchymal transition, EMT, eventually leading to invasion and metastases of the transformed epithelial cells [273]. As reviewed above, TRPV4 can possibly potentiate invasion of breast cancer cells through Ca^2+^-dependent activation of AKT that leads to changes in actin dynamics and downregulation of junctional E-cadherin [240,241]. Silencing or inhibition of TRPV4 by chemical compounds also clearly suppresses the migratory features of TRPV4-expressing 4T07 breast cancer cells, further confirming the role of TRPV4 in cancer metastasis [240]. The direct link between EMT and TRPV4 has been shown by other studies as well [191,192,274]. Furthermore, these studies revealed the connection of TRPV4 to the stiffness of the matrix, and TRPV4 itself has also been linked to the modulation of the ECM. TRPV4 is responsive to the biophysical changes of the surrounding matrix and can be triggered through signals transmitted through specific ECM polymers [46,47]. Consecutively, it can affect the remodeling of collagen in the ECM, affecting the mechanical features of the matrix [48,49]. In line with these observations, in breast cancer models, TRPV4 regulates ECM stiffness by affecting the expression of matrix proteins [241]. As fibrosis is very common in tumors, leading to tissue stiffening, such an environment creates a feedback loop through TRPV4 activity to further promote mechanical changes in the stroma. TRPV4 therefore seems to have a major role in several steps of the cancer progression through its mechanosensitive nature and ability to both respond and regulate the stiffness of the environment. Increased stiffness could also further alter the function of other mechanosensitive TRPV channels and at least TRPV6 has been shown to be responsive to elevated stiffness with increased expression in a breast epithelial cell model [45]. Increased levels of TRPV6 resulted in upregulation of EMT markers, possibly explaining the earlier findings on high TRPV6 levels in the invasive regions of mammary carcinomas [45].

### 5.3. Matrix Degradation

One of the features that makes cancer cells more invasive, is their ability to secrete matrix metalloproteinases (MMPs) through invadopodias, actin-rich cellular protrusions, to degrade the surrounding ECM [275]. Intriguingly, TRPs and Rho GTPase are also linked to the expression of MMPs. Rho GTPases and PI3K together with several other signaling factors regulate the formation of invadopodia core structure to enable MMP secretion [276,277]. Many of these players, regulating invadosome formation, are triggered in a mechanosensitive manner and MMP secretion is also dependent on the stiffness of the surrounding matrix [278,279,280]. Interestingly, the same signaling pathways (inc. PI3K/Rac1 pathway) that are responsible for MMP upregulation, also control TRPV2 trafficking to the plasma membrane [24,259,260,281,282,283,284]. Further, TRPV2 translocation is force-dependent and its expression leads to elevated levels of MMPs [226,259]. In contrast, depletion of TRPV2 by siRNAs led to lower levels of MMP-2 and MMP-9 in prostate tumors of nude mice xenografts [226]. It may be therefore hypothesized that stiffening of the matrix leads to TRPV2 translocation to the plasma membrane in a PI3K/Rac1-dependent manner and that TRPV2-mediated Ca^2+^- influx plays a role in MMP production through yet unknown mechanism, at least in prostate and ovarian cancer models [226,259,285]. Additionally, in bladder tumor models, high TRPV2 activity correlates with MMP-2 levels, playing an important role in the progression and invasion of this cancer type [232]. 

### 5.4. Angiogenesis

Besides matrix degradation, utilization of vasculature is extremely important for the spreading of cancer cells. Angiogenesis is a typical feature for the progressing tumors and the new vessels, induced by signaling within the cancerous tissue, are usually more fragile to allow intravasation of the invading cancer cells [286]. Mechanosensitive TRPV4 has been clearly linked with VEGF/VEGFR2 signaling and tumor angiogenesis [54,55], further indicating that TRPV4 can play a role in various processes that potentiate cancer cell invasion. Mechanical signaling, mediated by TRPV4, has been shown to regulate the vasculature: It is known to be essential for the shear-stress induced endothelial cell (EC) reorientation downstream of mechanosensitive integrins [47]. In tumor endothelial cells (TECs), TRPV4-mediated Ca^2+^-influx impacts actin dynamics and migration of TECs in a membrane-stretch-dependent manner [56]. In a lung carcinoma model, TECs seem to express lower TRPV4 levels and display abnormal mechanoresponse to ECM stiffness. This results in aberrant migratory features of the endothelial cells and TEC-promoted angiogenesis, consequently affecting tumor growth in a mouse model [57]. In contrast, stimulation of TRPV4 in the same model system leads to normalization of the vascular endothelium through restored mechanosensitivity and inhibits tumor growth [57]. It has also been noticed that such TRPV4-defective cells possess a lower expression of VE-cadherin, which possibly impacts the angiogenetic process and leads to vascular leakage [287]. Other than low TRPV4 levels, the upregulation of TRPV4 is also harmful to the intact endothelial junctions, as high TRPV4 activity in lung vasculature causes disruption of the intact endothelial walls [288]. This indicates that TRPV4 may possess both pro- and anti-angiogenic features, depending on the cancer type and genetic background or that up- or alternatively downregulation of TRPV4 impacts distinct mechanosensitive pathways.

### 5.5. Concerted Action of Various Biophysical Changes through TRPV Channels

**Figure 1:**
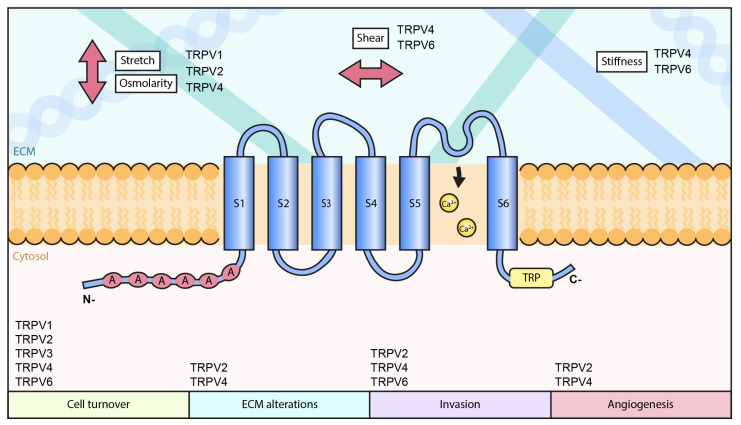
Hypothetical role of TRPV channels in cancer progression.

Other than the above mechanisms involved in cancer progression through mechanosensitive TRPV channels, TRPVs may play a role in cancer progression through several other yet unidentified ways. As cancer progression involves various biophysical changes in the extracellular environment, including stiffness, composition, hydrostatic pressure, compression and stretching, it is expected that all the mechanosensitive TRPV channels undergo some functional alterations. Combinations of various extracellular cues and signaling through several channels result in complex cellular phenotypes that may be difficult to mimic in in vitro assays. Besides the direct impact on the channel activity, transformation in other mechanosensitive structures that regulate TRPVs can take place. For instance, altered expression of cilia has been linked to the mechanical regulation of cancer progression [289]. TRPV channels that are directly linked to these structures and sensing force changes through them are clearly not able to respond normally to the mechanical signals. Additionally, other mechanosensitive structures, including integrin-based adhesion and the associated actin cytoskeleton can undergo various changes along cancer progression, also leading to changes in the activity of Ca^2+^-channels, either directly or indirectly. The possible association of distinct TRPV channels with cancer progression through their mechanosensitive features is summarized in Table 1 and a hypothetical model on the role of mechanosensitive TRPV channels in cancer progression is drawn in Figure 1.

## 6. Conclusions and Future Perspectives

Widely expressed mechanosensitive calcium channels are crucial mediators of physical signaling in various cellular processes and play a role in tumor pathophysiology. Alterations in cancer tissues, such as stiffness, composition, shear stress and pressure, act as physical stimuli that must impact the activity of these cationic channels. Although extensive studies have been performed on cancer-associated changes in mechanosensitive cell-adhesive and cytoskeletal structures, the membrane-embedded channels have obtained less attention and clearly require more studies in the future. What is still not well understood is how these mechanosensitive calcium channels handle forces, sensed through the plasma membrane, adhesive/cytoskeletal structures and the ECM fibers, as well as what extent of force changes are they dealing with. Many mechanisms that control f.i. cellular proliferation or apoptosis are extremely sensitive to modest changes in the Ca^2+^- homeostasis. Drastic changes in spatio-temporal calcium regulation through various channels in the transformed tissues may therefore reprogram cellular functions towards a more aggressive phenotype through several cascades. Although most of the TRP-mediated pathways involved in cancer progression are linked to the abnormal Ca^2+^ homeostasis, there are also indications for pore-independent functions for TRPs during metastatic development [290]. Clearly, this topic requires more studies in the future. One should also note that besides TRP channels, other mechanosensitive channels, like Piezo family channels, are important factors in sensing mechanical changes and in regulating mechanosensitive pathways that are linked to cancer progression [8,291,292]. These channels are also deregulated in various cancers and their cooperation with other ion channels along cancer progression should be investigated in the future.

Of the TRPV channel family, TRPV2 and TRPV4 have been mostly studied with respect to their altered mechanoresponsive features along cancer progression. However, it may well be that the other family members are as important in mediating abnormal mechanical signaling within transformed tissues. Hence, the mechanisms of how these distinct TRPV channels are responding to various biophysical changes in the tumor microenvironment, f.i. through gating or expression of the channel protein, needs more attention. Additionally, the intracellular signaling cascades downstream of these channels requires further studies and the exact mechanisms behind TRPV4-triggered alterations in contractile structures should be investigated. Moreover, the activation of the channels through direct and indirect mechanisms as well as the cross-talk in between these processes needs more attention. After all, these channels represent promising therapeutical targets that could be utilized in the management of advanced cancers.

## Figures and Tables

**Table 1:** Mechanosensitive TRPV channels in cancer progression.

Possible Role in Cancer Progression	Channel	Mechanical Activation	Mechanosensitive Pathways Involved	Cell or Cancer Type	References
**Migration**	TRPV2	Focal stimulation	Ca^2+^/PI3K/Akt/Rac1	Fibroblasts	[259,284]
**EMT**	TRPV4	Matrix stiffness	Ca^2+^/YAP/TAZ	NHEKs	[192]
**EMT**	TRPV4	Matrix stiffness	Ca^2+^/YAP/TAZ/PI3K/Akt	NHEKs	[191]
**EMT**	TRPV6	Stroma stiffness	Ca^2+^/CaMK	Breast epithelial cells	[45]
**Angiogenesis**	TRPV4	Matrix stiffness	VE-cadherin	WT/TRPV4KO mice	[287]
**Angiogenesis**	TRPV4	Matrix stiffness	Ca^2+^/Rho	CE cells	[57]
**Angiogenesis**	TRPV4	Stretch	Ca^2+^/PI3K/β1 integrin	CE cells	[47,199]
